# Supplementary material for: What does it take to learn the rules of RNA base pairing? A lot less than you may think
Source: bioRxiv. 2025 Aug 2:2025.07.31.668042. Preprint. [Version 1] doi: 10.1101/2025.07.31.668042 (PMC12324431; doi:10.1101/2025.07.31.668042)
Supplement: Supplement 1 [file media-1.gz › supplemental_material/src/rscape_v2.6.0.tar.gz!/rscape_v2.6.0/lib/infernal/Manuscripts/banded-cyk/figs/betavaried.pdf]

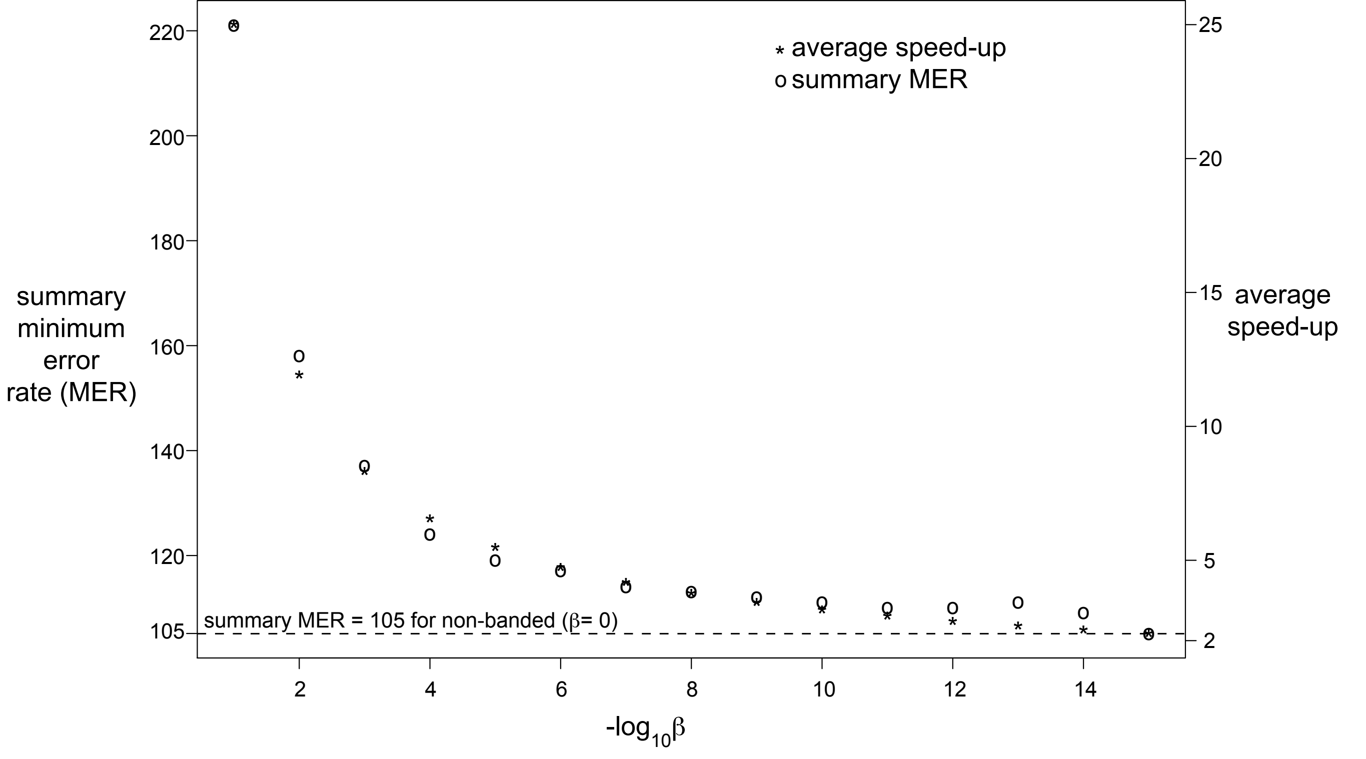

* average speed-up
o summary MER
summary minimum error rate (MER)
average speed-up
summary MER = 105 for non-banded (β= 0)
$-\log_{10}\beta$
220
200
180
160
140
120
105
25
20
15
10
5
2
2
4
6
8
10
12
14
